# Randomised crossover trial of neurally adjusted ventilatory assist (NAVA) for neonates with congenital diaphragmatic hernias: the NAN-C study

**DOI:** 10.1007/s00431-026-06888-5

**Published:** 2026-04-09

**Authors:** Sandeep Shetty, Allan Jenkinson, Grace Poole, Theodore Dassios, Chris Harris, Anay Kulkarni, Donovan Duffy, Anne Greenough

**Affiliations:** 1 Department of Neonatology, St George’s University Hospitals NHS Foundation Trust, London, UK; 2 City St George’s University of London, London, UK; 3 Department of Neonatology, King’s College Hospital NHS Foundation Trust, London, UK; 4 Department of Women and Children’s Health, School of Life Course and Population Sciences, Faculty of Life Sciences and Medicine, King’s College London, London, UK

**Keywords:** Neurally adjusted ventilatory assist (NAVA), Congenital diaphragmatic hernia (CDH), Mechanical ventilation, Neonate / Neonatal ventilation, Oxygenation index

## Abstract

Retrospective studies comparing NAVA to assist control ventilation (ACV) in neonates with congenital diaphragmatic hernia (CDH) have shown that ventilatory mode may improve respiratory parameters. The aim of this study is to determine if infants with CDH studied post-operatively had a lower oxygenation index (OI) on NAVA compared to ACV. This dual-centre randomised cross-over trial compared post-operative NAVA with ACV in infants with CDH. Infants were randomised to receive either NAVA or ACV first in a 1:1 ratio for a 4-h period. At the end of each 4-h period, blood gas analysis was performed and the OI calculated. The inspired oxygen concentration (FiO_2_), the peak inflation (PIP), and mean airway pressure (MAP) were averaged from the last 5 min on each mode. Eleven infants were randomised. Nine infants completed the trial**.** with median gestational age of 38 (range 34.6–39.3) weeks and median postnatal age of 7 (range 5–36) days. Eight had left-sided CDH, six had patch repair and two had thoracoscopic repair. The mean OI after 4 h on NAVA was 3.9 ± 1.8 compared to 5.9 ± 1.61 on ACV (*p* = 0.008). The peak Edi (6.05 ± 4.5 versus 9.86 ± 7.3 µV, *p* = 0.028), PIP (17 ± 6.3 versus 22 ± 5.3 cmH_2_O, *p* = 0.017), and MAP (8.7 ± 2.6 versus 11.1 ± 1.8 cmH_2_O, *p* = 0.008), expiratory tidal volume (5.06 ± 0.71 versus 9.86 ± 1.29 ml/kg, *p* = 0.043) were lower on NAVA versus ACV. Two infants were randomised, but the trial was stopped due to a low Edi signal.

*Conclusion*: NAVA compared to ACV improved oxygenation postoperatively in infants with CDH. On NAVA, infants had lower oxygen indices, peak Edi, expiratory tidal volume and peak and mean airway pressures.

**What is known:**

• *Neonates with congenital diaphragmatic hernia (CDH) require mechanical ventilation, but the optimal ventilatory strategy to minimise ventilator-induced lung injury remains unclear*.

• *Retrospective studies suggest that neurally adjusted ventilatory assist (NAVA) is feasible in CDH and may reduce ventilatory pressures and improve respiratory parameters*.

**What is new:**

• *This is the first prospective randomised crossover trial comparing NAVA with assist-control ventilation in postoperative neonates with CDH*.

• *NAVA significantly improved oxygenation (lower OI) and reduced peak and mean airway pressures, supporting more efficient ventilation at lower pressures.*.

## Introduction

Congenital diaphragmatic hernia (CDH) occurs due to an incomplete fusion of the diaphragm during foetal development, enabling abdominal viscera to herniate into the thoracic cavity [1]. Herniation may disrupt development of the lung and associated vasculature, resulting in pulmonary hypoplasia and pulmonary hypertension [2]. Neonates typically undergo surgical reduction in the first few days after birth [1]. The ventilation-perfusion mismatch often seen in infants with CDH can make the post-operative ventilation of this population challenging. The use of mechanical ventilation (MV) is the standard care for babies with CDH in neonatal intensive care units (NICU) across the UK [3]. The use of MV in this patient group, however, can injure the hypoplastic and contralateral lung, termed ventilator induced lung injury (VILI) [4]. The optimal mode of ventilation to prevent VILI in neonates with CDH remains unclear [5]. NAVA may trigger ventilatory support earlier in the respiratory cycle compared to pressure-triggered ventilatory methods (PTV), where the infant must initiate a sufficient change in pressure or flow to trigger ventilatory support [6]. Several small studies have demonstrated that NAVA improves patient-ventilatory asynchrony due to reduced trigger delays and auto or double triggering [7]. In the CDH population, it has been hypothesised that a structurally abnormal diaphragm may impede Edi signal detection and negate the benefits of NAVA. In a retrospective 1:2 matched case–control study, however, there was no significant difference in the Edi signal between infants with CDH ventilated with NAVA and those without [8]. In a retrospective cohort study, five out of seven infants that underwent a surgical patch repair for CDH had active Edi signals [7]. Several small studies have suggested NAVA is superior compared to assist control ventilation. A retrospective cohort analysis of 15 CDH neonates supported with 72 h of NAVA showed reductions in the peak inspiratory pressure (PIP), mean-airway pressure (MAP) and resulted in less sedative-medication use [9]. Another retrospective cohort of 12 infants in a single centre had d improvements in oxygenation index (OI) on NAVA, compared to pressure-support modes [10]. While results from retrospective analysis have been promising, to our knowledge there has been no prospective crossover trial investigating NAVA in infants with CDH. Our objective was to determine if in neonates with CDH, NAVA would result in a better OI, compared to ACV. The secondary objective was to determine if there were differences in other clinically important outcomes including sedative medication use.

## Methods

### Study design

NAN-C was a dual-centre, randomised, open-label cross-over trial designed with a superiority framework. It was carried out in two neonatal intensive care units (NICUs) in the UK, St George’s University NHS Foundation Trust (SGH) and King’s College Hospital NHS Foundation Trust (KCH). NAN-C was granted a favourable ethical opinion by the West of Scotland Research Ethics Committee (REC; reference number 23/WS/0070). NAN-C was prospectively registered on ClinicalTrials.gov NCT05839340 on May2023 [11].

### Participants

The study included neonates born with CDH. Eligible infants were identified by the researchers following discussion with the clinical team. Parents of participants meeting the screening inclusion criteria were approached to give consent for their infant’s participation. Infants were studied post-operatively.

At SGH, CDH infants were routinely ventilated using ACV on Servo-n ventilator (named ‘Pressure Control (PC)’ on the Servo-n ventilator, Maquet Critical Care, Solna, Sweden) which also offers the NAVA mode of ventilation.

At King’s College NHS Foundation Trust, infants were routinely ventilated using ACV on the SLE 6000 ventilator (software versions 4.3; SLE Ltd., South Croydon, UK). Hence, at KCH infants were transferred from the SLE 6000 to the Servo-n ventilator for the study and entered into a ventilatory support tolerance trial (VSTT).

During the VSTT infants were ventilated on ACV using the Servo-n ventilator (named ‘Pressure Control (PC)’ on the Servo-n ventilator, Maquet Critical Care, Solna, Sweden) for 1 h. During the VSTT, the positive end-expiratory pressure (PEEP) was kept between 4 and 5 cmH_2_O and inflation time at 0.36–0.4 s. The fraction of inspired oxygen concentration (FiO_2_) was adjusted with the aim of maintaining oxygen saturations between 85 and 95%. At this stage, infants requiring an FiO_2_ greater than 80% to maintain their oxygen saturation or requiring nitric oxide were excluded. If the infant passed the VSTT, they entered the study.

### Randomisation and allocation

**Fig. 1:**
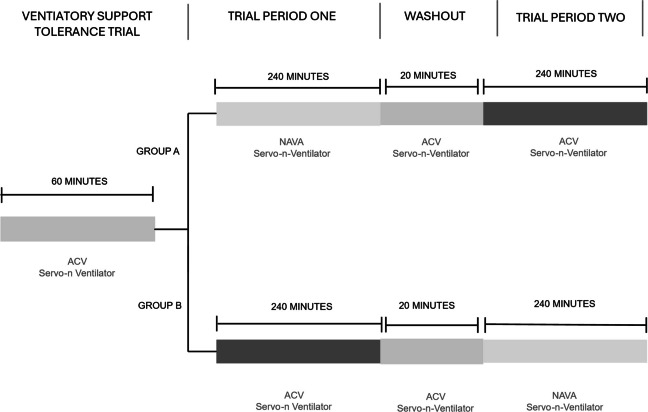
Study Protocol. At King’s College Hospital NHS Foundation Trust (KCH), infants are routinely ventilated using assist control ventilation (ACV) on the SLE 6000 ventilator. Upon entering the study, infants were transferred from ACV on the SLE 6000 to ACV on the Servo-n ventilator and underwent a ventilatory support tolerance trial (VSTT) for 60 min. If tolerated, infants were randomised to receive either ACV or neurally adjusted ventilatory assist (NAVA) for 4 h, followed by a 20-min wash-out period before commencing the alternate mode of ventilation (NAVA or ACV). At St George’s University Hospital (SGH), infants are routinely ventilated using ACV on the Servo-n ventilator. Upon entering the study, infants were randomised to receive either ACV or NAVA for 4 h, followed by a 20-min wash-out period before commencing the alternate mode of ventilation (NAVA or ACV)

Infants were randomised to receive either ACV or NAVA first for 4 h, followed by crossover to the alternative mode for 4 h. Randomisation was performed using a sequential opaque sealed envelope system (Fig. 1). The trial was open-label. Due to the nature of the intervention, clinicians were not blinded to ventilation mode. A 20-min stabilisation period (wash-out) was allowed following crossover to the second ventilation mode before outcome measurements were obtained.

### Interventions

At both sites, ventilation during the study was delivered using the Servo-n ventilator. Baseline ventilator settings and backup rates were maintained. In particular, the positive end expiratory pressure (PEEP) was kept at 4–5 cmH_2_O as had been used prior to the study and the inflation time was set, as previously, at 0.36 to 0.4 s. The apnoea time was set at 2 s and the upper pressure limit at least 5 cmH_2_O higher than the baseline settings but did not exceed 30 cmH_2_O. A six or eight French, 50 cm, Edi catheter was inserted and correct positioning confirmed as per the instructions of the manufacturer using the Edi catheter positioning guide function on the ventilator (Magnet Servo-n User Manual Version 4.6). The guide function displays the retrocardiac echocardiograph. Correct positioning was when the P waves and QRS complexes were visible in the uppermost leads and then decreased in size until the P waves disappeared in the lowest lead. Coloured highlighting of the central two leads appeared once the catheter was in the correct place. Once correct positioning was confirmed, the catheter was securely attached to the infant’s face using an adhesive dressing. Infants were then randomised to receive either ACV or NAVA first for 4 h and then to receive the alternative mode for the subsequent 4 h. Before the infant was changed to NAVA mode, the NAVA level was adjusted so that the displayed pressure waveform on NAVA closely matched the actual pressure waveform on the baseline settings, aiming for the peak Edi to be between 5 and 15 µV as per the recommendations of the manufacturer. The baseline ventilator settings were used to determine the backup settings to be used on NAVA in the absence of an Edi signal. The FiO_2_ was adjusted with the aim of maintaining oxygen saturations between 85 and 95%, as the target outlined in the CDH Euro Consortium 2015 [1].

### Outcomes

At the end of each 4 h, arterial or capillary blood gas analysis was performed and the oxygenation index (OI) calculated as the inspired oxygen concentration (FiO_2_) × mean airway pressure (MAP) × 100/PaO_2._ The SpO_2_/FiO_2_ (S/F) ratio was also calculated. The FiO_2_, the PIP, MAP, tidal volume and respiratory system compliance were recorded from the ventilator displays and averaged from the last 5 min of each 4-h period. The data were downloaded into Excel via a USB stick.

### Sample size

The planned sample size was 18 infants, as this would allow detection of a difference in oxygenation index between the two modes of one standard deviation, with 80% power and 5% significance. An interim analysis was planned to take place halfway through, i.e. after nine patients had completed the study. Studies with proportional assist ventilation (PAV), a ventilation mode which also provides tailored support throughout the infant’s inspiratory cycle, demonstrated the OI on PAV was better in all patients than on ACV [12, 13]. Similarly, mean OI after 1 h on NAVA was better than ACV [14]. In order to preserve the type I error at 5%, the interim analysis was conducted at 0.01 with the final analysis conducted using 0.04. This gave an overall type 1 error rate (significance level) of 5% [(1–0.01) × (1–0.04) = 0.95 = 1–0.05]. If the interim analysis showed *p* < 0.01, then the trial was to stop, and the final analyses conducted using the nine patients treated to that point.

### Statistical analysis

The results were positively skewed. Differences between ventilatory modes were assessed for statistical significance using the paired Wilcoxon signed-rank test using IBM SPPS statistical software, V.29 (IBM Corporation, USA). Geometric means and 95% confidence intervals were calculated manually in Excel for descriptive purposes. The ratio of geometric means and the corresponding 95% confidence intervals are presented descriptively and can be interpreted as the percentage difference between NAVA and ACV. To assess whether the order of ventilation influenced the magnitude of OI change, the differences in OI (NAVA – ACV) were compared between infants who received NAVA first versus ACV first using the Mann–Whitney *U* test.

## Results

Two randomised infants were withdrawn during VSTT due to persistently low Edi signals. Both infants had severe left-sided congenital diaphragmatic hernia, with pre-FETO observed-to-expected lung-to-head ratios (O/E LHR) of 13% and 15%, respectively. Both were male and underwent FETO at 26 weeks’ gestation. Gestational age at birth was 38.6 and 34.1 weeks, with corrected ages at the time of study of 39.6 and 36.4 weeks, respectively. Intraoperative findings demonstrated diaphragmatic agenesis in both cases: one infant had a type C defect (anteromedial and posteromedial rim present, with absent anterolateral and posterolateral rims), while the second had a type D defect, characterised by a completely deficient diaphragm and hiatus. Both infants had liver herniation.

**Fig. 2:**
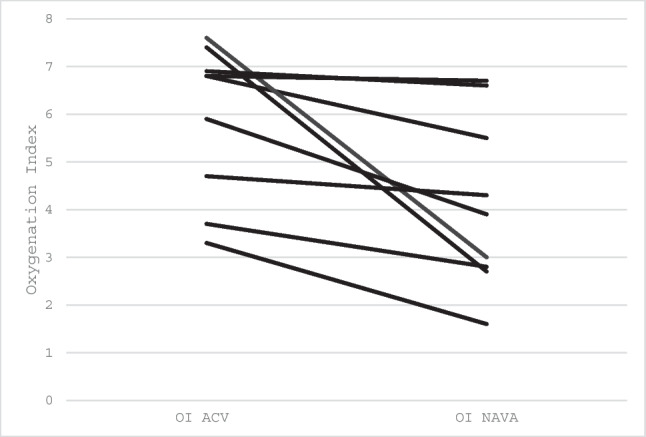
Individual OI data at the end of each mode

At the interim analysis, the comparison of oxygenation index (OI) on NAVA versus ACV was statistically significant. OI was lower on NAVA for all infants (Tables 1 and 2, Fig. 2). The nine infants, six males and three females, had a median gestational age of 38 weeks (range 34.6–39.3) and median birthweight of 3 kg (range 2.1–3.9). Infants were studied at a median postnatal age of 7 days (range 5–33). The median O/E LHR ratio was 46 (range 30–81). Eight infants had left-sided CDH; six underwent patch repair and two underwent thoracoscopic repair. Two infants underwent a FETO procedure at 26 weeks of gestation, with balloon deflation prior to birth. None of the infants had received antenatal steroids. Only one infant, born at 34 weeks received two doses of surfactant. Six infants received sedation, morphine infusion at maximum of 10 mcg/kg/hr; the concentration remained the same in both modes of ventilation and did not affect the Edi signal. Five infants were studied first on NAVA and four on ACV. There was no evidence that the order of ventilation affected the size of the OI difference (*p* = 0.66).

**Table 1:** Baseline characteristics of the study population (*n* = 9)

Male sex, *n* (%)	6 (67%)
Gestational age at birth, weeks	38.0 (34.6–39.3)
Birth weight, kg	3.0 (2.1–3.9)
Postnatal age at study, days	7 (5–33)
Left-sided CDH, *n* (%)	8 (89%)
Patch repair, *n* (%)	5 (56%)
Thoracoscopic repair, *n* (%)	2 (22%)
Observed/expected LHR	46 (30–81)
FETO performed, *n* (%)	2 (22%)
5-min Apgar score	9 (7–9)

**Table 2:** Results by ventilatory mode. The results are presented as the geometric mean (range) for each mode, the ratio of geometric means between the two modes and the corresponding 95% CI

	Mean PC	Mean NAVA	Ratio of geometric means (NAVA/ACV)	95% confidence interval for ratio	*p* value
Oxygenation index	5.9 (3.3–7.6)	3.9 (1.6–6.7)	0.66	0.50–0.90	0.008
Peak inspiratory pressure (cmH_2_O)	22 (14–28)	17 (8–26)	0.77	0.62–0.91	0.017
Mean airway pressure) (cmH_2_O)	11.05 (8–15)	8.65 (8–13)	0.78	0.68–0.89	0.008
FiO_2_	0.28 (0.22–0.35)	0.26 (0.21–0.36)	0.93	0.81–1.08	0.672
S/F ratio	355.7 (268.6–436.4)	412.5 (275–471.4)	1.16	0.95–1.26	0.203
Peak Edi (µV)	9.86 (1.2–27.0)	6.05 (1.4–16.7)	0.61	0.44–0.95	0.028
Expiratory tidal volume (ml/kg)	6.60 (5.21–7.9)	5.06 (4–5.98)	0.76	0.67–0.90	0.043
Compliance (ml/cmH_2_O)	1.31 (0.60–1.90)	1.46 (0.64–2.32)	1.11	0.84–1.49	0.345
Respiratory rate (breaths/min)	50.38 (40–64)	50.32 (42–65)	0.99	0.89–1.14	0.889
Oxygen saturations (%)	97 (94–99)	97 (94–99)	1.00	1.0–1.01	0.564

**Fig. 3:**
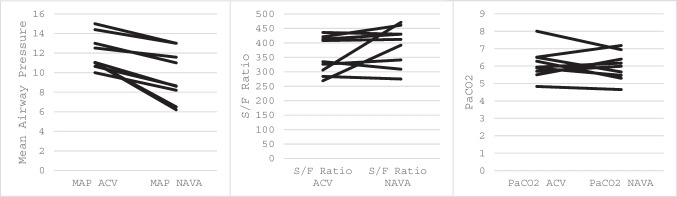
Secondary respiratory outcomes

The ratio of geometric means for OI, calculated manually for descriptive purposes, was 0.66 (NAVA/ACV), representing a descriptive 34% lower OI on NAVA compared with ACV (Table 2). Peak inspiratory pressure (PIP, *p* = 0.017) and mean airway pressure (MAP, *p* = 0.008) were significantly lower on NAVA (Fig. 3). Peak electrical activity of the diaphragm (peak Edi, *p* = 0.028) and expiratory tidal volume (*p* = 0.043) were also lower on NAVA. There were no significant differences between modes for FiO₂ (*p* = 0.672), S/F ratio (*p* = 0.203), compliance (*p* = 0.345), respiratory rate (*p* = 0.889) and oxygen saturation (*p* = 0.564).

## Discussion

In this prospective randomised crossover trial, in infants who completed the study NAVA was associated with a significantly lower OI compared with ACV in post-operative neonates with congenital diaphragmatic hernia (CDH). This improvement was observed consistently in all infants who completed the study and was accompanied by significantly lower peak inspiratory pressure (PIP) and mean airway pressure (MAP), suggesting more efficient oxygenation at lower ventilatory pressures. The SpO₂/FiO₂ (S/F) ratio was higher during NAVA compared with pressure control ventilation, although this difference did not reach statistical significance. This may reflect the limitations of the S/F ratio when oxygen saturations are already high due to the plateau of the oxyhaemoglobin dissociation curve [15]. Two infants with severe left sided CDH did not progress beyond the VSTT due to low Edi suggesting reduced diaphragmatic signal associated with severe diaphragmatic agenesis.

Beck et al. showed that NAVA improves neuromechanical efficiency by unloading the diaphragm while maintaining spontaneous respiratory drive, as evidenced by reduced Edi amplitude without suppression of breathing effort [16]. Similar reductions in diaphragmatic effort during NAVA have been demonstrated in neonates and children using oesophageal pressure–Edi relationships, supporting the concept that lower Edi during NAVA reflects effective unloading rather than diaphragmatic dysfunction [17, 18]. Our finding of lower peak Edi on NAVA is consistent with these observations and suggests improved diaphragm–ventilator coupling in infants with CDH. NAVA delivers breath-by-breath proportional assistance, which may be particularly advantageous in CDH, where respiratory drive and mechanics can fluctuate substantially in the post-operative period.

Concerns have previously been raised that structural abnormalities of the diaphragm in CDH, particularly following patch repair, might impair the detection or reliability of the Edi signal and limit the utility of NAVA. However, both our findings and prior retrospective studies suggest that clinically usable Edi signals are achievable in the majority of CDH infants, including those undergoing patch repair [5, 8, 19]. In our cohort, NAVA was feasible in nine of eleven infants studied, supporting its applicability in this population. This is despite infants receiving sedation at the same dose throughout the study, which did not influence the results.

Previous CDH studies of NAVA have largely been retrospective and observational. Kallio et al. reported reductions in PIP, MAP, and sedative exposure in CDH infants supported with NAVA [20], while Gentili et al. demonstrated the feasibility of NAVA during weaning in a small cohort [21]. In addition, Amin and Arca reported the successful use of non-invasive NAVA following CDH repair in a retrospective series, supporting feasibility across the post-operative respiratory care pathway, although this was reported in abstract form only [19]. Our study extends these findings by providing prospective, randomised crossover data, thereby reducing confounding related to disease severity and inter-patient variability. Unlike earlier retrospective reports, our trial demonstrates that improvements in oxygenation occur rapidly and consistently within hours of initiating NAVA, supporting a direct physiological effect rather than delayed clinical improvement.

This study has limitations. The sample size was small, and the trial was stopped early following a statistically significant interim analysis, which may overestimate treatment effects. The short exposure period limits conclusions regarding long-term outcomes such as duration of ventilation or chronic lung disease. In addition, the study was open-label and investigators were not blinded to ventilation mode, which may introduce potential bias. Nonetheless, the crossover design strengthens internal validity by allowing each infant to act as their own control; there was no evidence that the order of ventilation influenced the magnitude of OI change. As the same ventilator was used for each mode, the significant differences demonstrated are due to the differences in the modes, rather than differences in the ventilator performance. The infants included had a wide range of severity including patch repair, prematurity and range of postnatal age when repaired, yet we saw a positive effect of NAVA in all infants. We used capillary blood samples to calculate the OIs in three patients. We used the same method for both ventilation modes the end of each of the 4 h periods, thus the use of capillary blood sampling did not bias our results. The infants were all clinically stable when assessed and none were seriously ill, had shock, hypotension or peripheral vasoconstriction at the time of assessment [22]. Thus, we feel it was appropriate to calculate the OIs from the capillary blood samples.

In conclusion, NAVA significantly improved oxygenation despite lower airway pressures in post-operative neonates with CDH. Larger multicentre trials are required to determine whether these short-term physiological benefits translate into improved long-term clinical outcomes.

## Data Availability

No datasets were generated or analysed during the current study.

## Abbreviations

ACV: Assist-control ventilation
CDH: Congenital diaphragmatic hernia
Edi: Electrical activity of the diaphragm
FETO: Fetoscopic endoluminal tracheal occlusion
FiO₂: Fraction of inspired oxygen
MAP: Mean airway pressure
MV: Mechanical ventilation
NAVA: Neurally adjusted ventilatory assist
NICU: Neonatal intensive care unit
O/E LHR: Observed-to-expected lung-to-head ratio
OI: Oxygenation index
PAV: Proportional assist ventilation
PC: Pressure control
PEEP: Positive end-expiratory pressure
PIP: Peak inspiratory pressure
PTV: Pressure-triggered ventilation
VILI: Ventilator induced lung injury
VSTT: Ventilatory support tolerance trial
